# Stereotactic body radiotherapy with carbon ions as local ablative treatment in patients with primary liver cancer

**DOI:** 10.1186/s13014-025-02594-y

**Published:** 2025-02-18

**Authors:** Paula Hoffmeister-Wittmann, Philipp Hoegen-Saßmannshausen, Livia Wicklein, Fabian Weykamp, Katharina Seidensaal, Christoph Springfeld, Michael T. Dill, Thomas Longerich, Peter Schirmacher, Arianeb Mehrabi, René Michael Mathy, Bruno C. Köhler, Jürgen Debus, Klaus Herfarth, Jakob Liermann

**Affiliations:** 1 Department of Radiation Oncology, Heidelberg University Hospital, Heidelberg, Germany; 2 Heidelberg Institute of Radiation Oncology (HIRO) and National Center for Radiation Research in Oncology (NCRO), Heidelberg, Germany; 3 National Center for Tumor Diseases (NCT), NCT Heidelberg, a partnership between DKFZ and Heidelberg University Hospital, Heidelberg, Germany; 4 Clinical Cooperation Unit Radiation Oncology, German Cancer Research Center (DKFZ), Heidelberg, Germany; 5 Department of Medical Oncology, Heidelberg University Hospital, Heidelberg, Germany; 6 Liver Cancer Center Heidelberg, Heidelberg, Germany; 7 Department of Gastroenterology, Infectious Diseases, Intoxication, Heidelberg University Hospital, Heidelberg, Germany; 8 German Cancer Research Center (DKFZ) Heidelberg, Research Group Experimental Hepatology, Inflammation and Cancer, Heidelberg, Germany; 9 Institute of Pathology, Heidelberg University Hospital, Heidelberg, Germany; 10 Department of General, Visceral & Transplantation Surgery, Heidelberg University Hospital, Heidelberg, Germany; 11 Department of Diagnostic and Interventional Radiology, Heidelberg University Hospital, Heidelberg, Germany; 12 Heidelberg Ion Beam Therapy Center (HIT), Heidelberg, Germany; 13 German Cancer Consortium (DKTK), Partner Site Heidelberg, Heidelberg, Germany

**Keywords:** Hepatocellular carcinoma, SBRT, Cholangiocarcinoma, Local control, Carbon ion radiotherapy, Hypofractionation

## Abstract

**Background and aims:**

Liver cancer is the third leading cause of cancer related death due to treatment resistance and late onset of symptoms (Rumgay in J Hepatol 77: 1598–1606, 2022). The role of external beam radiotherapy (EBRT) in treatment of unresectable liver cancer needs to be defined. The use of particle therapy such as carbon ion radiation therapy (CIRT) with high linear energy transfer (LET) could increase efficacy of EBRT while limiting the toxic effects of radiation on non-cancerous liver tissue. Promising effects of CIRT have been described in several studies during the past decades, mostly in Japan. To date, no standardized treatment protocol has been established and European data on CIRT for liver cancer is lacking. This retrospective analysis aims to investigate efficacy and safety of hypofractionated CIRT compared to photon-based stereotactic body radiation (SBRT) in primary liver cancer.

**Method:**

Thirty-six (n = 36) and twenty (n = 20) patients with primary malignant liver tumors were treated with hypofractionated CIRT (4 fractions) and photon-based SBRT, respectively, between 2011 and 2022 and were retrospectively evaluated for survival, local control, and toxicity.

**Results:**

Two-year local control rate after CIRT was 92.3%. Compared to photon- based SBRT, CIRT scores with a significantly longer median distant progression free survival (3.1 versus 0.9 years). In a matched pair comparison of the two treatment regimens, the CIRT cohort demonstrated both longer 2-year overall survival (100% versus 59.6%) and longer 2-year distant PFS (75.7% versus 22.9%). No significant impairment of liver function was observed in either cohort.

**Conclusion:**

In this retrospective analysis, patients who received CIRT presented excellent local tumor control and had better oncologic outcomes than patients who received photon-based SBRT. SBRT with carbon ions is a promising local ablative treatment option that needs further investigation in large prospective trials.

**Supplementary Information:**

The online version contains supplementary material available at 10.1186/s13014-025-02594-y.

## Introduction

Liver cancer is a heterogenous group of malignancies, of which hepatocellular Carcinoma (HCC) is the most prevalent primary liver cancer, followed by intrahepatic cholangiocarcinoma (iCCA) [2]. Most cases of HCC occur on the basis of chronic liver inflammation and cirrhosis with hepatitis B virus (HBV)- or hepatitis C virus (HCV) infection as well as steatotic liver disease (SLD) such as alcohol-associated liver disease (ALD) and metabolic dysfunction associated steatotic liver disease (MASLD) as major risk factors for cancer development [3]. The decision for the appropriate therapeutic option of HCC usually depends on the Barcelona Clinic Liver Cancer (BCLC) classification which includes tumor size, performance status and liver function [4]. In early stages, surgical resection, liver transplantation and ablation are curative treatment options. However, only approximately 30% of all HCC cases are eligible for potentially curative resection due to liver dysfunction or comorbidities [5]. In unresectable stages, local tumor control via locoregional therapies such as transcatheter arterial chemoembolization (TACE), radiofrequency ablation (RFA) and microwave ablation (MWA) has been proven to increase patients’ survival [6, 7]. ICCA however is a growing subgroup of cholangiocarcinoma (up to 75% of all CCA cases) arising from bile ducts within the liver and can be subdivided into small and large bile duct iCCA [8, 9]. Among others, risk factors for development of CCA are Primary Sclerosing Cholangitis (PSC), liver cirrhosis or viral hepatits [10]. In a non-metastatic tumor stage, surgical resection can be a curative treatment option. Patients not suitable for surgery have to face limited treatment options since evidence for treatment efficacy of locoregional treatments is restricted [11]. Very rarely, mixed tumors that have features of both HCC and CCA form a distinct entity: HCC-CCA tumors [12]. Those tumors present an aggressive biology associated with poor prognosis.

The role of external beam radiotherapy (EBRT) in treatment of unresectable primary liver cancer has yet to be defined. By introduction of stereotactic body radiation (SBRT), safety and efficacy of EBRT have been drastically improved but application of photon-based radiotherapy is mostly limited by the risk of radiation-induced liver disease (RILD) and bowel toxicity [13, 14]. Therefore, it is critical to apply a dose of radiation that is effective enough to target the tumor while sparing healthy liver tissue. However, comparison of photon based SBRT followed by systemic sorafenib treatment compared to sorafenib treatment only, showed improved oncological outcomes for addition of SBRT without enhanced toxicities [15]. The use of particle therapy such as proton beam therapy (PBT) and carbon ion radiation therapy (CIRT) with high linear energy transfer (LET) allows for an increase in relative biological effectiveness (RBE), limiting the effects of radiation on normal liver tissue while enabling dose escalation within target lesions. Compared to PBT, CIRT scores with a higher RBE and a steeper lateral dose penumbra which enables an even more precise radiation beam path. Even though promising effects of CIRT for HCC treatment have been described in several studies during the past decades, standardized treatments are lacking in international guidelines. In 2021, Abousaida et al. reviewed recent retrospective and prospective studies of CIRT and PBT in HCC and found that CIRT may be particularly beneficial for patients with HCC lesions > 10 cm because of its minimal impact on liver function [16]. More recently, final results of a clinical phase-I study (PROMETHEUS) using a treatment scheme of up to 4 × 10.5 Gy (RBE) have been published, showing a objective response rate of 80% after 27.3 months of follow-up in a selected cohort of patients suffering from HCC [17]. Data for application of CIRT for treatment of iCCA is even more limited, since only one retrospective analysis exists. Kasuya et al. described treatment with normofractionated CIRT for iCCA in a cohort of 56 patients with a 2-year local control rate of 58.2% [18]. Taken together, data favoring application of CIRT in the context of treating primary liver cancer is promising, yet limited and needs further investigation. The present study analyzes efficacy and safety of hypofractionated CIRT for both HCC and iCCA compared to photon-based SBRT in a single-center retrospective trial.

## Material & methods

### Study design

Patients with primary liver malignancy who were treated with hypofractionated CIRT at Heidelberg University Hospital between 2011 and 2022 were analyzed retrospectively. Patients who were treated within the prospective PROMETHEUS trial were excluded from this analysis. Local Control (LC), overall survival (OS), progression free survival (PFS) as well as adverse events (AE) were evaluated. LC was defined as no evidence of tumor regrowth within the planning target volume. Patients were matched for sex, age (± 5 years), tumor entity and size of lesion (± 2 cm) with a cohort that received photon-based SBRT. Of each treatment modality, 17 patients could be included in the matched pair analysis. This study was performed following institutional guidelines and the Declaration of Helsinki of 1975 in its most recent version. Ethical approval for the study was given from the local ethics committee of the medical faculty of the University of Heidelberg (S-042/2023) and the institutional review board waived the requirement for written informed consent from each individual.

### Patient characteristics

Between 2011 and 2022, 36 patients were identified who were treated with four fractions of CIRT due to a malignant liver tumor at the University Hospital of Heidelberg. Among those, 32 patients with confirmed diagnosis of HCC, 3 patients with iCCA and one patient with mixed HCC/CCA were included. In the same time frame, 20 patients with primary liver cancer (16 patients with HCC, 4 patients with iCCA) received photon-based SBRT. Indication and recommendation for SBRT was approved in an interdisciplinary tumor board consisting of board-certified specialists from pathology, oncology, gastroenterology, radiation oncology, radiology and surgery. Basic patient and treatment data were collected from the local radiation oncology registry. Clinical, operative, and hospital course records were reviewed.

### Treatment and follow up

In all patients receiving CIRT, RT was carried out with one or two beams. Irradiation with carbon ions was exclusively performed using active raster-scanning with daily image guidance via CT imaging. Photon-SBRT was performed using intensity-modulated arc radiotherapy (mostly using 2 arcs) with daily (mostly 4D) conebeam CT image-guidance at Elekta Versa HD. For both modalities, patients were immobilized in supine position with vacuum cushion immobilization and abdominal compression or less frequently deep inspiration breath-hold (DIBH). Contrast-enhanced computed tomography (CT) scans including arterial, venous, and native phases as well as native 4D-CT (3-mm slice thickness) were used for treatment planning. Additionally, contrast-enhanced magnetic resonance imaging (MRI) was used for target volume delineation. CIRT treatment planning was conducted using Syngo PT Planning version 13 (Siemens®, Erlangen, Germany) or Raystation (RaySearch Laboratories, Stockholm, Sweden). The clinical target volume (CTV) included the visible tumor on contrast-enhanced CT or MRI (gross tumor volume or GTV) with a margin of 5 mm for subclinical spread of disease. Based on the performed 4D-CT an internal target volume (ITV) was delineated that included respiratory movement of the CTV. For the planning target volume (PTV), an additional isotropic margin of 5 mm (CIRT: 7 mm in beam direction) was added to account for positioning uncertainties. CIRT was performed in 4 fractions and was applied every other day. Median duration of radiotherapy was 7 days (range 7–11 days); RBE was calculated using the LEM I model. Photon-based SBRT was performed in a median of 8 fractions in consecutive days; BED was calculated using an α/β/-ratio of 10 Gy.

In our institution, imaging follow-up included a contrast-enhanced abdominal MRI or a CT scan 6–8 weeks after completion of treatment and every 3–6 months within the first 2 years after radiotherapy. Follow-up visits were timed with the same frequency and included clinical examinations and registration of treatment-related toxicities.

### Statistics

All statistical analyses were performed using GraphPad PRISM® 10.1.1 (GraphPad). OS and local and distant PFS (L- / D-PFS) were calculated using Kaplan–Meier analysis. OS was calculated as the time from the start of RT to death or the date of last follow-up. D-PFS was defined as the time from the start of RT to tumor progression at a site other than the primary tumor or death, whereas L-PFS included only local tumor progression at the primary tumor lesion or death. Patients without tumor progression, or patients who were lost to follow-up were censored. Results are expressed as mean, range, and percentage. Subgroups were compared using the log-rank test. *p*-values of 0.05 or less were considered statistically significant. Odds ratios accompany 95% confidence intervals. Association between patients’ characteristics and OS or PFS respectively assessed as a multivariate analysis using the Cox proportional hazard model. Differences in cohort characteristics were estimated using either χ^2^ or Fisher’s exact test for categorical data and unpaired t-tests for parametric data. Treatment response was categorized according to mRECIST. Observed adverse events were evaluated from patients’ medical records and classified according to version 5.0 of the Common Terminology Criteria for Adverse Events (CTCAE). Analysis of the matched pair cohort was performed using paired t test (parametric data) or Wilcoxon test (non-parametric data).

## Results

### Patient and tumor characteristics within the CIRT cohort

The median age was 71 years (Q1 63 years; Q3 79 years), with 72% male and 28% female patients. Fifty percent of the patients had prior therapy, of which 50% received TACE, 38% surgical resection, 22% RFA (Radiofrequency Ablation) and 5% each SIRT (selective internal radiotherapy), IRE (irreversible electroporation) and MWA (microwave ablation), respectively. Median size of irradiated lesions was 3.5 cm (Range: 1.3–9.6 cm). Median CTV and PTV were 49.8 cm^3^ (range 8.7–502.1 cm^3^) and 141.2 cm^3^ (range 36.6–919.6 cm^3^), respectively. Mean healthy liver dose was 7 Gy RBE (range 2.6 – 16.3 Gy RBE). Among the treated HCC patients, most patients had a tumor in BCLC stage C (52%), followed by BCLC stage A (42%). Fifty percent of all patients had cirrhosis, of whom 78% had CHILD Pugh Score A and 22% had CHILD Pugh Score B cirrhosis. Chronic Hepatitis C (HCV) and Hepatitis B (HBV) was known in 27% and 15% of the patients. Detailed patient- and tumor- and treatment characteristics are shown in Tables 1, 2, and 3.

**Table 1:** Patient characteristics (n = 36)

	Patients	%
*Gender*		
Female	10	28%
Male	26	72%
*ECOG*		
0	18	50%
1	17	47%
2	1	3%
*Liver cirrhosis (CHILD)*		
No	9	25%
A	23	63.9%
B	4	11.1%
*Origin of cirrhosis*		
Alcohol	10	37.1%
Hepatitis C	4	14.8%
Hepatitis B	7	25.9%
Nutritional	3	11.1%
Cryptogenic	3	11.1%
	Median	Range
Age	71	28 – 88
BMI	28.1	16.1 – 38.7

**Table 2:** Tumor characteristics (n = 36)

	Patients	%
Tumor stage	33	92%
HCC	19	58%
BCLC A	12	36%
BCLC B	2	6%
BCLC C	3	8%
CCA	2	66.7%
UICC I-II	1	33.3%
UICC III-IV		
Histology	21	58.3%
Yes	15	41.7%
No		
AFP elevation	2	5.5%
Yes	34	94.5%
No		
Liver lobe	14	38.9%
Left lobe	19	52.8%
Right lobe	3	8.2%
Both lobes affected		
Tumor lesions in total	22	61.1%
1	6	16.7%
2	8	22.2%
≥ 3

**Table 3:** Treatment characteristics (n = 36)

	Patients	%
Dose levels ([Gy] RBE)	6	16.7%
32.4	10	27.8%
35.2	4	11.1%
38	8	22.2%
40	8	22.2%
42		
CIRT target lesions	33	91.7%
1	2	5.5%
2	1	2.8%
3		
Prior treatment	17	47.2%
Any	9	25%
TACE	7	19.4%
Resection	4	11.1%
RFA	1	2.8%
IRE	1	2.8%
SIRT	1	2.8%
MWA		
	Median	Range
Total Dose ([Gy] RBE)	38	32.4 – 42
GTV (cm^3^)	24.9	1.5 – 393.2
CTV (cm^3^)	49.8	8.7 – 502.1
ITV (cm^3^)	78.7	4.3 – 678.5
PTV (cm^3^)	141.2	36.6 – 919.6
Diameter of lesion (cm)	3.4	1.3 – 9.6

### Oncological outcomes

**Fig. 1:**
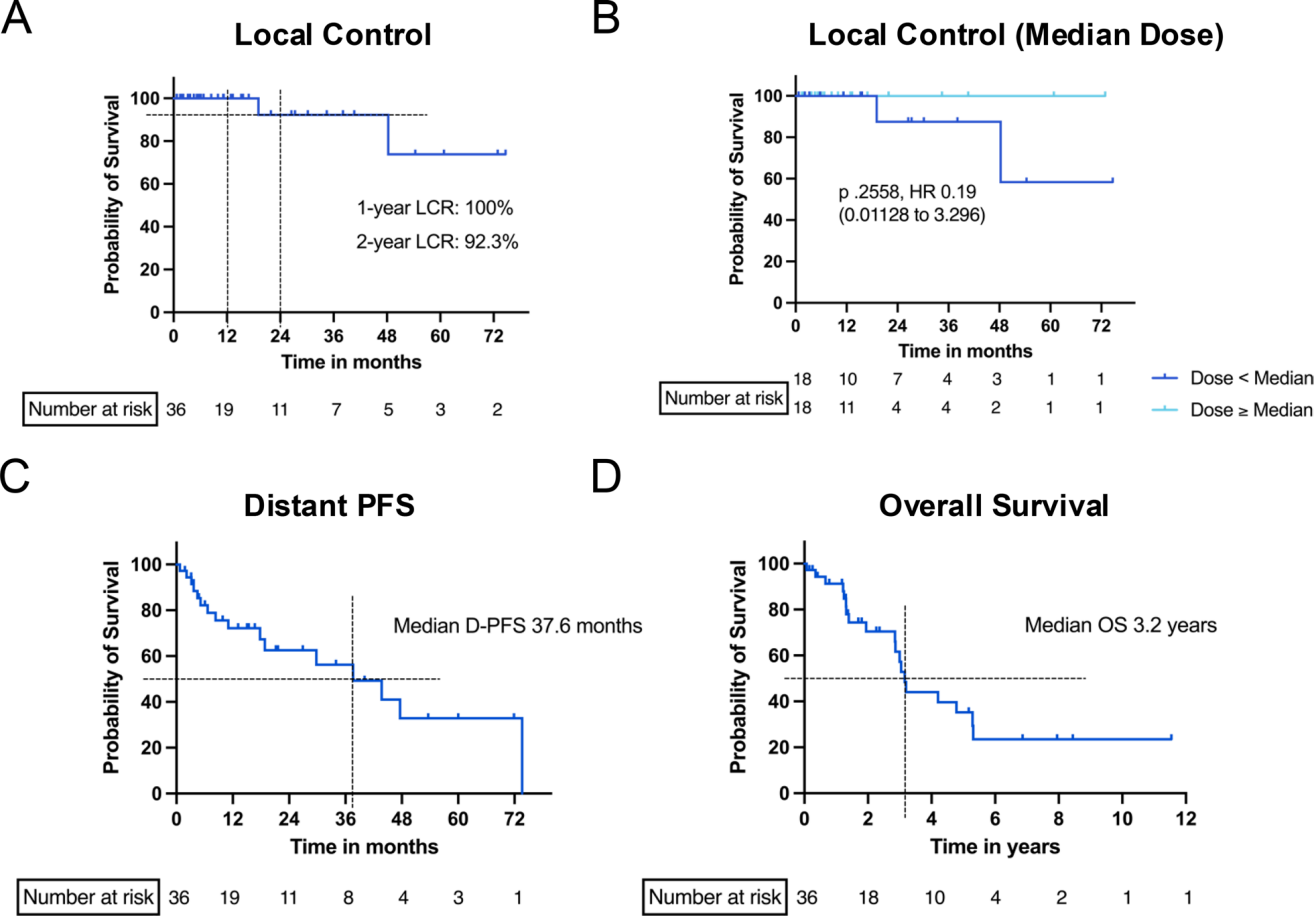
Kaplan-Meyer analysis of local control, D-PFS and overall survival after CIRT. 1- and 2-year local control rate were 100% and 92.3% respectively **(A)**, separation of the cohort after median total dose revealed a 100% Local control rate of patients receiving ≥ median total dose of 38 Gy RBE (**B**). Distant progression free survival was 37.6 months (**C**) and overall survival was 3.2 years (**D**)

The median total dose of CIRT was 38 Gy RBE (Range: 32.4–42 Gy RBE), divided into 4 single doses every other day. One- and 2-year LC rate (LCR) were 100% and 92.3% respectively (Fig. 1A). Patients receiving ≥ median dosages of radiation had a LCR of 100% (Fig. 1B). After a median follow up of 19.5 months (range 3.2–137.2 months) only two patients experienced local progression of the irradiated lesion. Both of these patients suffered from HCC and were irradiated with a total dose of only 35.2 Gy RBE (4 × 8.8 Gy RBE) and received an additional CIRT upon progress on the same lesion without additional toxicity. One of the patients had another local progression within a year, whereas the other patient has been stable since. Median D-PFS was 37.6 months (Fig. 1C). Overall Local Treatment Response Rate (PR, CR) was 64.8%. Median OS was 3.2 years (range 0.15–11 years, Fig. 1D). An univariate cox proportional hazard model showed a significant association of OS with sex (better OS for male patients, *p* = 0.0344, Table 4). No correlation of clinicopathological characteristics and D-PFS could be seen (Table S1).

**Table 4:** Univariate analysis for OS (n = 36)

	Median	HR (95% CI)	*P*
Age	71	1.38 (0.52–3.5)	0.5067
Sex (f / m)		0.38 (0.12–1.21)	0.0344
BCLC (A-B / C)		1.14 (0.41–3.2)	0.7954
T (1 / 2–3)		0.59 (0.23–1.53)	0.2905
BMI (kg/m^2^)	28.1	0.69 (0.27–1.81)	0.4405
Max. diameter (cm)	3.4	0.91 (0.33–2.51)	0.8546
Cirrhosis (yes/no)		0.64 (0.24–1.73)	0.3488
Liver lobe (right/left)		0.43 (0.16–1.14)	0.0700
Prior treatment (yes/no)		0.82 (0.31–2.14)	0.6750

### Photon-based SBRT cohort

Between 2014 and 2022, 20 patients with primary liver cancer (16 HCC, 4 CCA) received photon-based SBRT with a total median BED of 83 Gy divided into 8 fractions (BED range 48–150 Gy in total, divided into 3–10 fractions). The most common prescription was 50 Gy in 5 fractions, prescribed to the 80% isodose. Median size of irradiated lesions was 3.2 cm (Range: 1.4–6.8 cm). Median CTV and PTV were 74 cm^3^ (range 19.5–639.7 cm^3^) and 135.4 cm^3^ (range 42.6–799.7 cm^3^), respectively. Liver cirrhosis was present in 85%, of which 88% were classified as CHILD A cirrhosis. After a median follow-up of 17 months (range 1–54.9 months) only one patient (CCA) experienced local progression within the radiation field. Detailed patient characteristics are listed in Table S2–S4. Univariate analysis of clinicopathological data revealed a significant correlation between survival and BMI level with better survival rates with BMI ≥ median with better overall survival for patients being slightly overweight (BMI 24.8–39.6) (Table S5).

### Photon-based SBRT versus CIRT

**Fig. 2:**
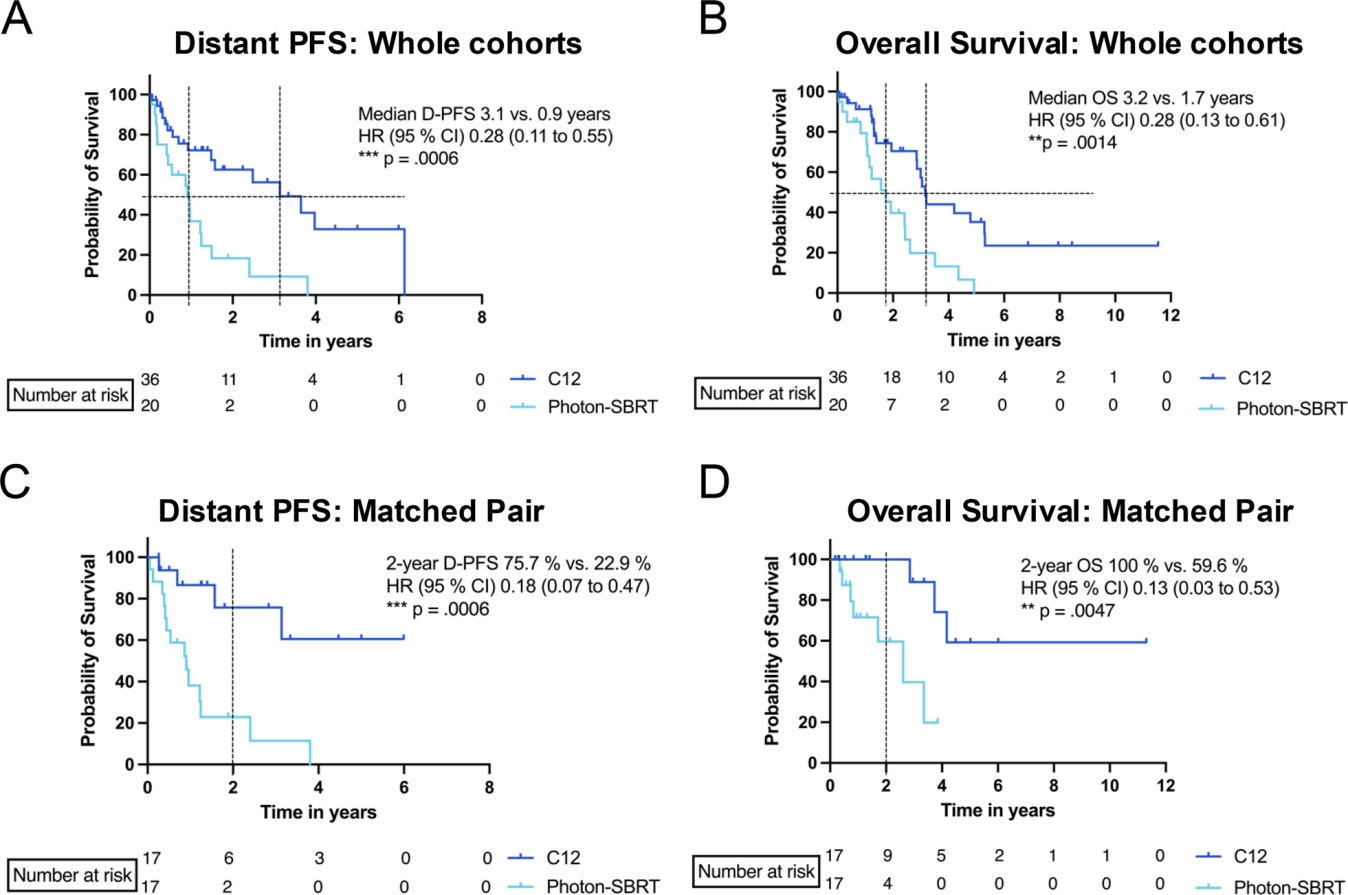
Kaplan-Meyer analysis of overall survival and distant progression free survival in the matched-pair analysis. Comparison of CIRT and Photon-SBRT for distant progression free survival (PFS) and Overall survival between the whole cohorts (**A**, **B**) or as matched-pair analysis (**C**, **D**)

Comparison of oncological outcomes in the two cohorts revealed a significant better D-PFS for CIRT versus photon-based SBRT (Fig. 2A, 3.1 versus 0.9 years, *p* = 0.006). Overall survival was significantly better for patients receiving CIRT compared to Photon-SBRT (Fig. 2B, 3.2 versus 1.7 years, *p* = 0.0014). 2-year local control rate was comparable (92.3% for CIRT versus 95.2% for photon-based SBRT). No significant differences in patient-, tumor- or treatment characteristics could be observed (Table 5). For a more profound analysis, a matched pair analysis between two cohorts was performed. Patients were matched for sex, age (± 5 years), tumor entity and size of lesion (± 2 cm). 17 patients of each treatment modality could be included in the matched pair cohort. Overall survival (2-year OS 100% versus 59.6%, *p* = 0.0398) and D-PFS (2-year D-PFS 75.7% versus 22.9%, p = 0.0006) were significantly better for the CIRT cohort in both cases (Fig. 2C, D).

**Table 5:** Characteristics

	CIRT (n = 36)	Photon-SBRT (n = 20)	*p*-value
	n (%)	n (%)	
Gender			0.7488
Female	10 (28%)	5 (25%)	
Male	26 (72%)	15 (75%)	
ECOG			0.0811
0	18 (50%)	8 (40%)	
1	17 (47%)	10 (50%)	
2	1 (3%)	2 (10%)	
Liver cirrhosis (CHILD)			0.1771
No	9 (25%)	3 (15%)	
A	23 (64%)	15 (75%)	
B	4 (11%)	2 (10%)	
Tumor stage			0.0729
HCC	33 (92%)	16 (80%)	
BCLC A	19 (58%)	8 (50%)	
BCLC B	12 (36%)	6 (37.5%)	
BCLC C	2 (6%)	2 (12.5%)	
CCA	3 (7%)	4 (20%)	
UICC I-II	2 (66.7%)	2 (50%)	
UICC III-IV Tumor lesions in total	1 (33.3%)	2 (50%)	
1	22 (61.1%)	13 (65%)	*0.6255*
2	6 (16.7%)	5 (25%)	
≥ 3	8 (22.2%)	2 (10%)	
	Median (Range)	Median (Range)	
GTV (cm^3^)	24.9 (1.5 – 393.2)	24 (5.2 – 333.8)	*0.7071*
CTV (cm^3^)	49.8 (8.7 – 502.1)	74 (19.5 – 639.7)	*0.1760*
Age	71 (28 – 88)	73 (44 – 88)	*0.5873*
BMI (kg/m^2^)	28.1 (16.1 – 38.7)	24.7 (18.8 – 39.6)	*0.2489*
Diameter of lesion (cm)	3.4 (1.3 – 9.6)	3.2 (1.4 – 7)	*0.7498*

### Toxicity

No dose-limiting toxicities were reported in either cohort. In the CIRT cohort, 73 adverse events (AEs) grade I—III occurred, distributed among 78% (28/36) of all patients. In the Photon-SBRT cohort, toxicities grade I—III occurred in all patients, totaling 59 AEs (Table 6). No significant difference in frequency of grade I / II or III toxicities could be observed in-between cohorts (*p* = 0.4923, χ^2^ = 1.417). The most common grade I radiation-related toxicity in both cohorts was fatigue (22.5% versus 11.6%). All cases of anemia, leukopenia and thrombocytopenia occurred either as long-term toxicity—most likely due to subsequent systemic treatments or on the basis of pre-existing cytopenia. All patients who were found to have grade III toxicities had these attributes prior to initiation of radiotherapy hence no therapy-related grade III toxicities occurred in neither CIRT nor Photon-SBRT cohort. Of note, liver function was not affected by radiation (no Grade > 2 elevation of liver enzymes, no > Grade 1 Ascites, no change in Child Pugh Score). Remarkably, within the CIRT cohort, four patients received re-radiation within 32.4 months (range 8.2–50.6 months) following the same protocol, of which two patients even had the same target volume. Again, no dose limiting toxicities and no significant impairment of liver function could be observed. Overall, both treatment regimen showed only slight differences in toxicities and did not cause persistent toxicities.

**Table 6:** Toxicity

	CIRT (n = 36)	Photon–SBRT (n = 20)
	Patients (%)	Patients (%)
**Grade I**	**49 (67.1%)**	**43 (73%)**
Abdominal pain	2 (4.1%)	–
Diarrhea	2 (4.1%)	2 (4.6%)
Fatigue	11 (22.5%)	5 (11.6%)
Radiodermatitis	1 (2%)	–
Weight loss	–	2 (4.6%)
Nausea/Vomiting	–	1 (2.3%)
Aszites	–	3 (7%)
Leukopenia	3 (6.1%)	2 (4.6%)
Anemia	6 (12.2%)	7 (16.3%)
Thrombocytopeniac	7 (14.3%)	7 (16.3%)
Increased INR	3 (6.1%)	5 (11.6%)
Elevated liver	2 (4.1%)	4 (9.3%)
Enzymes	2 (4.1%)	–
GOT	3 (6.1%)	1 (2.3%)
GPT	2 (4.1%)	–
gGT	5 (10.2%)	4 (9.2%)
AP		
Bilirubine		
**Grade II**	**17 (23.3%)**	**13 (22%)**
Abdominal pain	1 (5.9%)	–
Fatigue	2 (11.8%)	2 (15.4%)
Leukopenia	3 (17.6%)	2 (15.4%)
Anemia	3 (17.6%)	–
Thrombopenia	2 (11.8%)	7 (53.9%)
Elevated liver	–	1 (7.7%)
Enzymes	1 (5.9%)	1 (7.7%)
gGT	4 (23.5%)	–
Bilirubine	1 (5.9%)	–
Nausea / Vomiting	1 (5.9%)	–
Radiodermatitis		
**Grade III**	**7 (9.6%)**	**3 (5%)**
Anemia	–	2 (66.7%)
Leukopenia	3 (43.9%)	–
Thrombopenia	4 (57.1%)	1 (33.3%)

## Discussion

Liver cancer is the third leading cause of cancer related death, creating an urgent need for effective yet tolerable locoregional therapies [19]. In this retrospective study, we could show that Carbon ion radiation therapy is an effective treatment option for locally advanced primary liver cancer with limited toxicity. Regardless of pre-existing liver cirrhosis, liver function was not significantly impaired in any patient, even though the median follow-up interval of 19.5 months is considerably short to evaluate late toxicities. The 2-year OS rate of our CIRT cohort was 100% and a 2-year local control rate of 92.3% which is in line with previous studies [20–22]. After a follow-up period of 36 months, data were only available for a few patients due to death or loss to follow-up, hence survival data at later time points might not be conclusive. In our study, distant progression free survival and overall survival were significantly better for patients receiving CIRT compared to patients receiving photon beam SBRT in a matched-pair analysis, which might be due e.g. to different immunomodulatory effects of CIRT compared to SBRT [23]. No significant differences in patient, tumor or treatment characteristics were found that could have influenced survival outcomes. Nevertheless, the selection of patients for the different treatment modalities may have influenced the results emphasizing a prospective evaluation of both treatment modalities. Due to the retrospective nature of this analysis, information on adverse events was extracted from patients' medical records and may therefore be biased.

The feasibility of CIRT has been evaluated in several previous studies, mostly within a cohort of Asian patients. In Japan, hypofractionated treatment for HCC using 48–60 Gy (RBE) in 4 fractions showed favorable LC and survival rates [20]. When comparing carbon ion treatment data from Japan, one has to consider different RBE-models for calculating the biological dose. While most Asian centers use the mixed beam model for passive scattering or the microdosimetric kinetic model (MKM), the local effect model (LEM) is most widely used in Europe [24]. Steinstraeter et al. reported conversion factors in order to make comparison easier: Following their recommendations, the aforementioned 48–60 Gy would equal 38–44 Gy within the LEM I model [25]. Since the local control rate is higher in patients receiving more than 38 Gy (RBE) in total, this dose should be targeted for future applications. In view of the limited toxicity rates, especially considering the liver function in this retrospective analysis, one could argue for a less restrictive approach to the indications. Tumors located near the central hepatobiliary tract or tumors located on the surface of the liver must be irradiated carefully due to possible toxicity. Results from Dawson et al. also suggested, that size of the lesion should not limit radiation indications, since even a total diameter of 20 cm did not lead to > grade 2 AEs [15].

Just recently, promising results of phase I PROMETHEUS study even revealed a 100% local control rate of hypofractionated CIRT using the biological RBE-model LEM I and active raster scanning for HCC in a small cohort of 20 patients [17]. Carbon ion radiation for intrahepatic cholangiocarcinoma has only been evaluated in one retrospective analysis of 56 patients: Within the J-CROS study, patients were treated with a median dose of 76 Gy (RBE; $\cong$ 83 Gy after LEM I) in 20 fractions, with a 2-year Local Control Rate of 58% [18]. To our knowledge, no recommendations for SBRT treatment regimens have been described and radiotherapy in general plays a minor role in treatment of primary liver cancer. Most patients receive systemic treatment including both chemo- and immunotherapy. Due to treatment toxicities as well as pre-existing organ dysfunctions, some patients are not eligible for systemic therapies raising the need of effective local treatment options.

European guidelines include thermal ablation or transarterial chemoembolization (TACE) for irresectable HCC BCLC stage A and B respectively [26, 27]. SBRT is mentioned as alternative treatment for irresectable BCLC A stage only. A prospective randomized trial (TRENDY) revealed better 2-year local control rates for SBRT compared to TACE with drug-eluting beads (100% versus 43.6%) but the study had to be closed due to slow accrual [28]. In a propensity score matched review comparing CIRT and TACE in 477 patients for treatment of naive, single-tumor HCC, OS as well as LC and PFS were superior in the CIRT cohort [29]. Recommendations for treatment of primary liver cancer of the American Society for Therapeutic Radiology and Oncology (ASTRO) include proton-based SBRT or IMRT. Different fractionation schemes are mentioned, even though most schemes consist of 3–5 fractions with a total dose of 30–60 Gy depending on the liver function [30, 31]. Recommendations for carbon ion radiation in the context of primary liver cancer are completely lacking in all European and American guidelines.

In order to embed CIRT in the therapeutic tree for treating primary liver cancer, prospective studies are necessary. Particularly for HCC studies comparing efficacy and toxicity of CIRT with MWA, RFA or TACE in different settings are needed. For CCA prospective studies evaluating efficacy and safety of CIRT is needed since only retrospective data is available.

## Conclusion

This study provides retrospective evidence that hypofractionated CIRT is a safe and potent treatment option for patients with primary liver cancer. Our data suggest that the use of carbon has a favorable effect on PFS and overall survival compared to photons. However, this requires further exploration to determine a potential basis for these findings. Due to its low integral radiation dose to the remaining liver tissue, CIRT could widen the therapeutic window of liver irradiation for patients with severely impaired liver function.

## Supplementary Information

Additional file1.

## Data Availability

All data generated or analyzed during this study are included in this published article.

## Abbreviations

CTCAE: Common terminology criteria for adverse events
(i)CCA: (Intrahepatic) cholangiocarcinoma
AE: Adverse events
ALD: Alcohol-associated liver disease
ASTRO: American Society for Therapeutic Radiology and Oncology
BCLC: Barcelona clinic liver cancer
BMI: Body mass index
CIRT: Carbon ion radiation therapy
CR: Complete remission
CT: Computed tomography
CTV: Clinical target volume
D- / L- PFS: (Distant- / local) progression free survival
DIBH: Deep inspiration breath-hold
EBRT: External beam radiotherapy
GTV: Gross tumor volume
HBV: Hepatitis B virus
HCC: Hepatocellular carcinoma
HCV: Hepatitis C virus
IMRT: Intensity modulated radiotherapy
IRE: Irreversible electroporation
ITV: Internal target volume
LC: Local control
LCR: Local control rate
LEM: Local effect model
LET: Linear energy transfer
MASLD: Metabolic dysfunction associated steatotic liver disease
MKM: Microdosimetric kinetic model
MRI: Magnetic resonance imaging
MWA: Microwave ablation
OS: Overall survival
PBT: Proton beam therapy
PR: Partial remission
PTV: Planning target volume
RBE: Relative biological effectiveness
RFA: Radiofrequency ablation
RILD: Radiation-induced liver disease
RT: Radiotherapy
SBRT: Stereotactic body radiation
SIRT: Selective internal radiotherapy
SLD: Steatotic liver disease
TACE: Transcatheter arterial chemoembolization
