# Supplementary material for: Stereotactic body radiotherapy with carbon ions as local ablative treatment in patients with primary liver cancer
Source: Radiat Oncol. 2025 Feb 18;20:23. doi: 10.1186/s13014-025-02594-y (PMC11834390; doi:10.1186/s13014-025-02594-y)
Supplement: Supplementary file 1 — Additional file1. [file 13014_2025_2594_MOESM1_ESM.docx]

**Supplementary Material:**

**
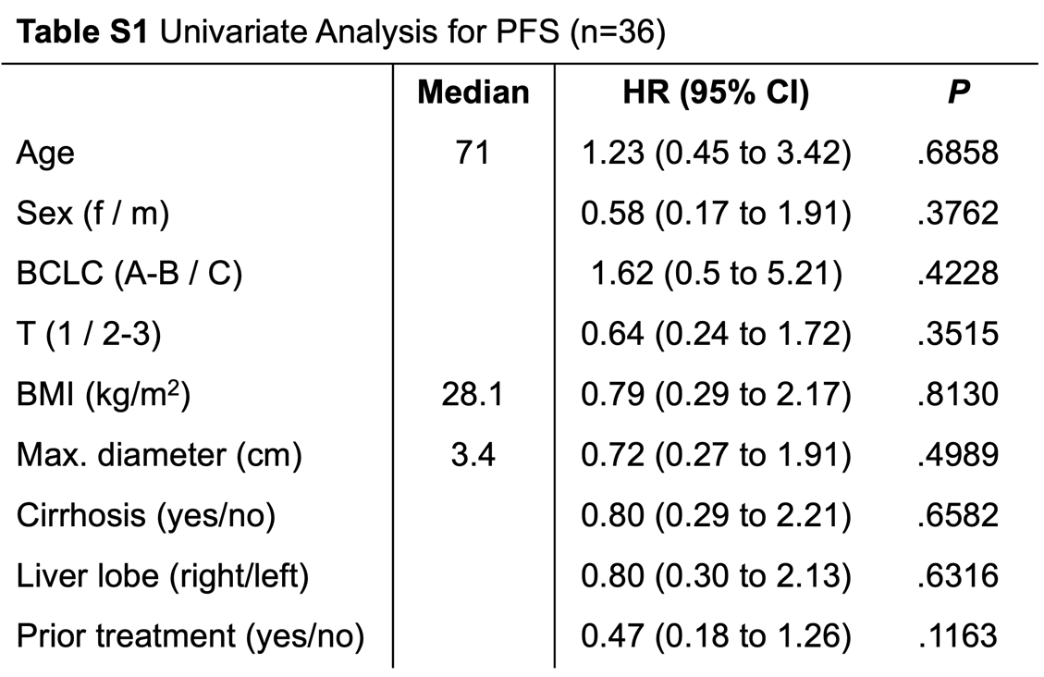
**

**Table S1: Univariate Analysis of clinicopathological data and distant PFS (CIRT).** No significant correlation between patient characteristics and dPFS could be observed.

| 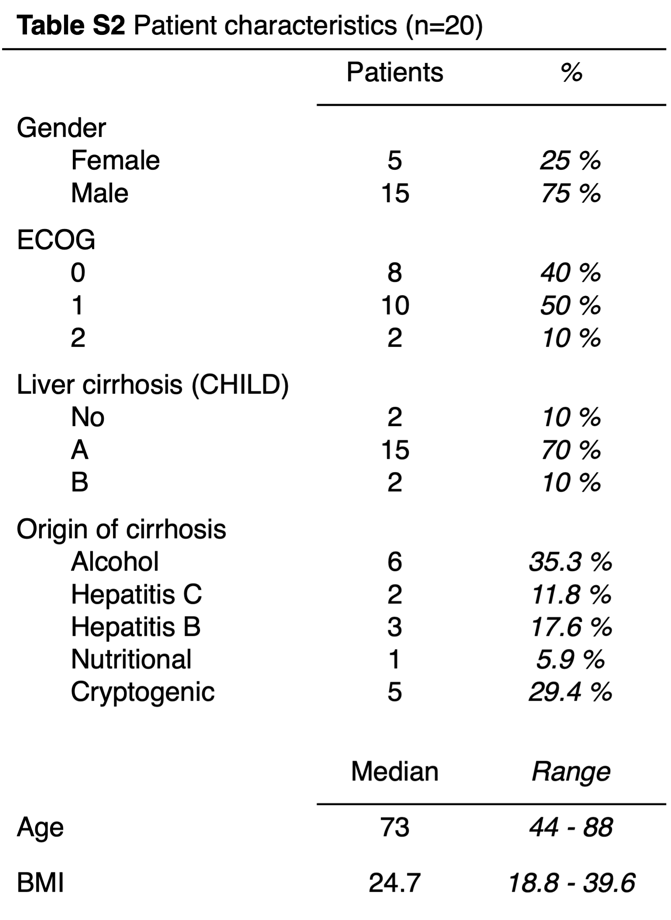 | 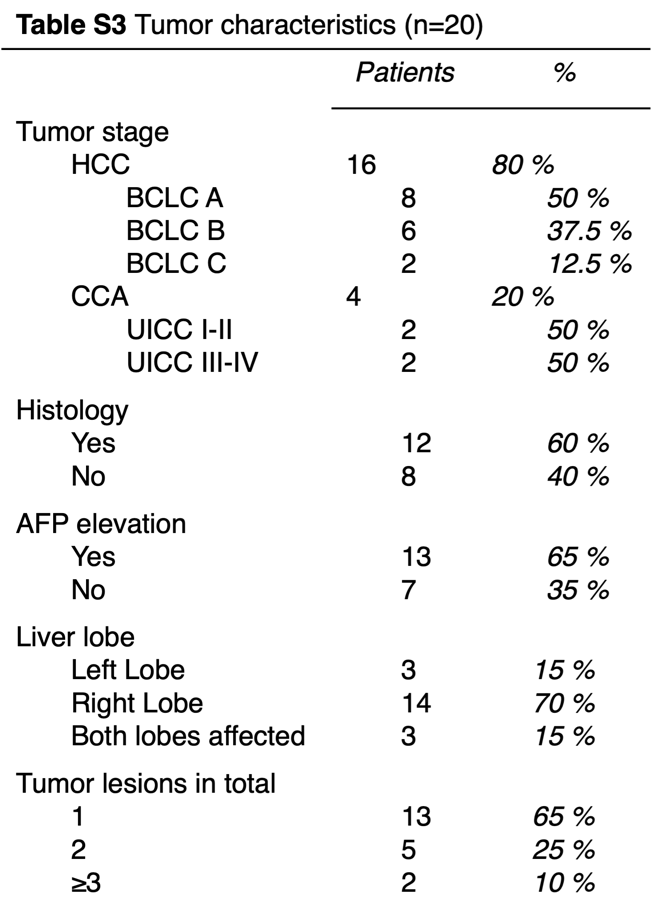 |
| --- | --- |
| **Table S2 and S3: Patient characteristics (S2) and Tumor Characteristics (S3) of the Photon-SBRT cohort.** | |

**
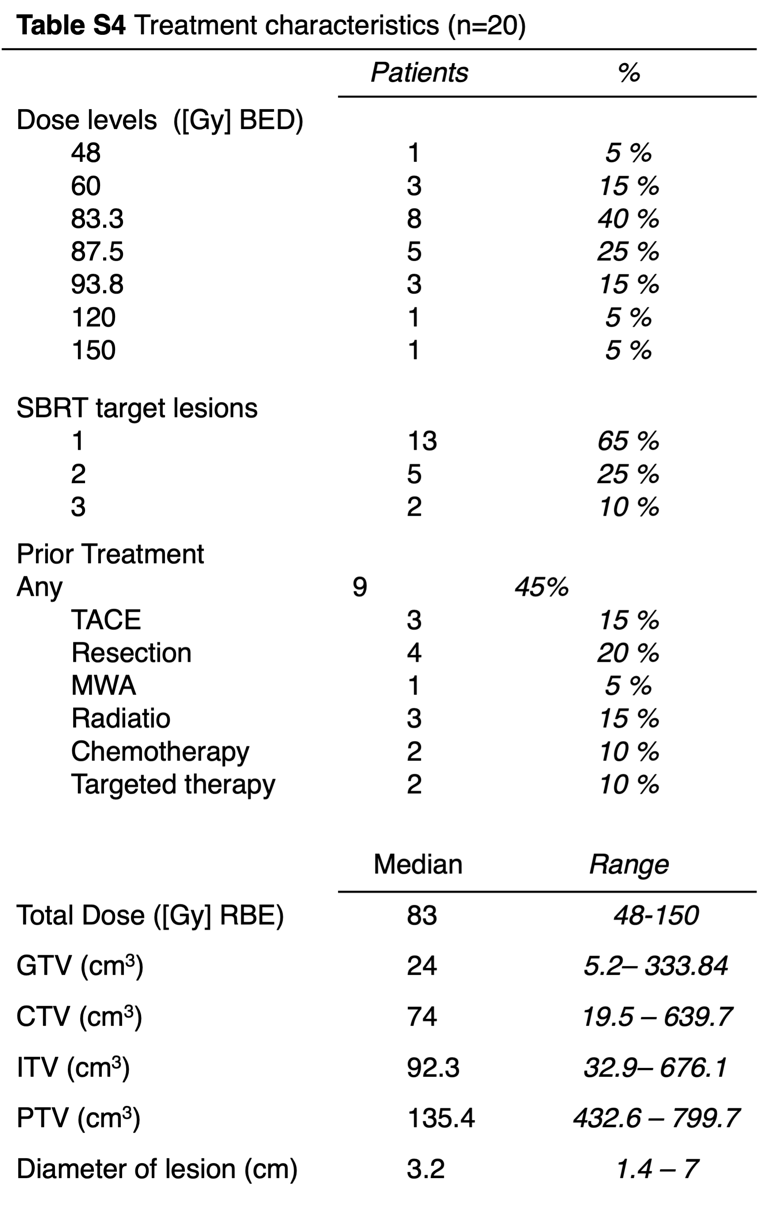
**

**Table S4: Treatment characteristics of the Photon-SBRT cohort.**


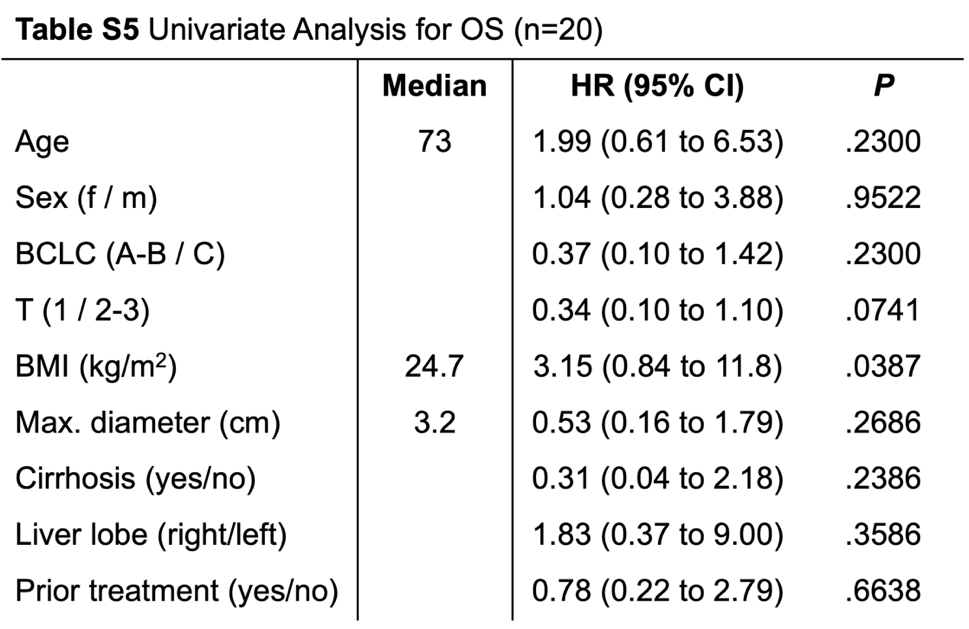


**Table S5: Univariate Cox hazard analysis of clinicopathological data (Photon-SBRT).** Significant better survival for patients with BMI ≥ median.
